# Evaluation of the usefulness and understandability of information leaflets on fall prevention from the perspective of hospital patients and their relatives

**DOI:** 10.1111/hir.12531

**Published:** 2024-04-30

**Authors:** Eva Maria Lissa Pock, Christa Lohrmann, Magdalena Hoffmann, Christine Maria Schwarz, Daniela Schoberer

**Affiliations:** ^1^ Institute of Nursing Science, Medical University of Graz Graz Austria; ^2^ Executive Department for Quality and Risk Management University Hospital Graz Graz Austria; ^3^ Division of Endocrinology and Diabetology, Department of Internal Medicine Medical University of Graz Graz Austria; ^4^ Research Unit for Safety and Sustainability in Health, c/o Division of Plastic, Aesthetic and Reconstructive Surgery, Department of Surgery Medical University of Graz Graz Austria

**Keywords:** evaluation, older people, patient information

## Abstract

**Background:**

Falls are a major problem among adults over 60 years. Multiple preventive measures must be taken. Written information leaflets can support the knowledge transfer and positively influence recall of the information provided.

**Objective:**

The aim was to ensure usefulness and understandability of the information leaflets on home fall prevention from the target groups' perspective.

**Methods:**

A cross‐sectional survey study with a feedback questionnaire for patients and relatives was conducted at a university hospital in Austria. Quantitative data analysis and qualitative content analysis according to Schreier were performed.

**Results:**

The majority (63.9%) of patients rated the overall impression as “very good”. 44.2% of the relatives rated it as “very good” and 23% as “good”. The question “appealing design” was the only one with a statistically significant difference between patients and relatives. Subgroup analysis has shown a statistically significant difference between educational groups regarding the questions “easy to read” and “easy to understand”.

**Conclusion:**

It could be shown that the information leaflets were already well tailored to the target group. The few comments regarding understandability were considered to improve the content of the information leaflets. A further evaluation regarding the benefit of the fall prevention leaflets in discharge management should be performed.

**Key Messages**

- Information leaflets that are systematically developed and use plain language are seen as useful and understandable from patients' and relatives' perspectives.
- Patients and relatives want detailed information on fall prevention strategies with concrete examples and tips for safety measures in information leaflets.
- To provide clearer and more understandable information leaflets for older people and their relatives, the target group should be involved in the development and evaluation process.
- The findings increased knowledge regarding which information older people and their relatives want to be included in an information leaflet on fall prevention.
- A structured process that includes the target group is valuable for the development of information materials addressing care problems.

## BACKGROUND

Life expectancy has increased over the last decades; therefore, the number of people over 60 years of age has grown (Eurostat, 2021). As a person ages, their susceptibility to disease increases, their musculature weakens and, consequently, their mobility decreases. These age‐related changes represent risk factors for falls (Niccoli & Partridge, 2012; Pasquetti et al., 2014). Falls are a major health problem among adults over 60 years of age worldwide. Between 20% and 30% of those who fall suffer injuries, a deterioration in mobility, and/or reduced independence (World Health Organization, 2021).

According to the World Health Organization, a fall is an event that results in a person coming to rest inadvertently on the ground, floor or other lower level (World Health Organization, 2021).

Many people who have fallen require medical care and – especially in the case of older people – often long‐term care, depending on their injuries. This can place an enormous cost burden on the health care systems (Gazibara et al., 2017; World Health Organization, 2021). Falls happen mostly due to interactions among several factors; the more risk factors there are present, the higher the risk of falling (Prüfer‐Storcks, n.d.; Sibley et al., 2014).

To prevent falls, the individual risk factors must be identified (Callis, 2016), and adequate preventive measures must be taken (Lee et al., 2013; Park, 2018; World Health Organization, 2021). In addition to multifactorial fall prevention strategies that may include physical exercises, a medication review, or environmental adaptations (Hopewell et al., 2018; Hopewell et al., 2020; Schoberer, Eglseer, et al., 2018), educational interventions play an important role as well (Lohrmann et al., 2019). Educational measures are most effective if they are provided in the context of a multifactorial programme (Lee et al., 2014). Studies have shown that it is helpful to provide written, IT‐based or user‐oriented information leaflets to patients upon their discharge from hospital (Heng et al., 2020; Newnham et al., 2017). Many patients forget some of the information they receive from health care professionals in hospital due to anxiety, their older age, or an overload of information or are unable to reproduce it correctly (Nguyen et al., 2019; Wolderslund et al., 2020). The patients' recall of this information is influenced by their self‐assessment of the importance of the information they have received (Wolderslund et al., 2020). Written information leaflets have been shown to improve health knowledge and positively influence recall of the information provided (Lin et al., 2014). Written information leaflets can be particularly effective if they are tailored to meet the needs of the target group. Scientific information must be worded in such a way that is understandable to the target group, and the layout should be clear (Centers for Medicare & Medicaid Services, 2010a; Wizowski et al., 2014). Health changes, such as altered vision or deterioration of cognitive processes, negatively affect the understandability of information leaflets. Easily recognizable headings, a large font, short and clear sentences and a high‐contrast colour design must be considered when creating information leaflets for older people (Centers for Medicare & Medicaid Services, 2010b).

The discharge conversation can be used to make patients aware of their risk of falling and to inspire them to change their behaviour (Hill et al., 2011; Ott, 2018). When giving information on fall prevention, relatives should be involved (Cuesta‐Benjumea et al., 2018). They can adjust the living space, point out hazards and work together to develop strategies in order to prevent falls (Wilkinson et al., 2018). Information about disease and care presented in plain language can help relatives support persons in need (Cuesta‐Benjumea et al., 2018; Wizowski et al., 2014).

To ensure the usefulness and understandability of written information leaflets, the material must be evaluated by the target group (van Beusekom et al., 2018; Wizowski et al., 2014). Studies that evaluated information leaflets show that the information is not always understandable for the target group even if the leaflet is produced according to current, evidence‐based guidelines. The readers have difficulties absorbing uncertain evidence, whereas clear tips on how to overcome the problem are easier to understand (Lins et al., 2011; Schmitz et al., 2010).

A guide for developing information leaflets developed by Wizowski et al. (2014) provides a framework that comprises evaluation as a key aspect. This guide recommends obtaining feedback from the target group to determine the usefulness and understandability of the information leaflet. One common method used to obtain feedback is to conduct surveys (Wizowski et al., 2014). According to Wizowski et al. (2014), the evaluation should involve both patients and their relatives. Leaflets on fall prevention in nursing and care homes for older persons were adapted for use in ‘REDACTED’ hospitals. These should be used in addition to verbally providing information about home fall prevention to older people at risk during the discharge conversation.

The study was carried out to assess the usefulness and understandability of information leaflets from the perspectives of patients and their relatives.

## METHODS AND MATERIALS

### Study design and setting

The study design is a cross‐sectional survey. This survey was performed to investigate the usefulness and understandability of the information leaflets on home fall prevention from the patients' and relatives' perspectives at a medical university hospital in Graz, Austria.

### Participants' recruitment and sampling

The study was conducted at a university hospital Graz, Austria. The participating Department of Neurology and the Department of Dermatology and Venerology were chosen by the nursing manager at the hospital. The ward managers of the participating departments were informed of the study by a researcher and passed this information on to the nursing staff. Convenience sampling was used to select participants (patients and relatives). Patients who were admitted to the hospital as inpatients during the survey period from March to May 2021 or relatives of these patients who met the inclusion criteria were invited to participate in the survey. The nursing manager permitted a timeframe of three months for data collection. We aimed at recruiting 180 participants, based on the experience of prior projects at this hospital as well as on data from the literature regarding response rates (Horevoorts et al., 2015). However, the final sample size was determined by how many patients and relatives could be recruited during this period. To meet the inclusion criteria, patients needed to be over 65 years of age and to display at least one risk factor for falls according to the evidence‐based guideline on fall prevention (Schoberer, Findling, et al., 2018). These especially included patients who had a history of falls (in the last six months) and/or at least one disease‐related risk factor (dementia, impaired vision or incontinence problems) for falls and/or had been prescribed fall‐inducing medications (sedatives, anticonvulsants or antipsychotics). Relatives had to be caregivers of patients at risk of falling who were being treated as inpatients at the respective departments.

### Data collection

As part of the discharge management, nursing staff verbally provided patients and relatives with information about falls, and this was supported by the written information leaflet for fall prevention in nursing and care homes for older people. In this context, the feedback questionnaire was handed out to the participants by the nursing staff. Participants were informed that participation was voluntary and that nonparticipation would not result in any disadvantages regarding their treatment or care. Participants were given time to read the information leaflet and then to complete the feedback questionnaire. The completed feedback questionnaire was put into an unlabelled envelope by the participants and dropped into an available box on the wards.

### Information leaflets on fall prevention

The information leaflets on fall prevention for patients and relatives referred to in this paper were developed by the Institute of Nursing Science at the Medical University of Graz and the Executive Department for Quality and Risk Management, University Hospital Graz, Austria. These are shown in the supporting information (Figures S1 and S2). The content is based on international guidelines. A systematic literature review was conducted to identify these guidelines in advance. The search for relevant literature was carried out in specific guideline databases, PubMed and Google Scholar. The authors limited their search to the last ten years and set a language restriction to the German, English, Spanish, French and Portuguese languages. The search with MeSH terms, keywords and synonyms resulted in the identification of 141 potentially relevant guidelines. Title, abstract and full‐text screenings were then carried out based on predefined inclusion and exclusion criteria. The application of these criteria enabled the authors to narrow this pool of relevant literature down to three remaining guidelines. These were appraised using the appraisal tool AGREE II (Brouwers et al., 2019). Based on the quality assessment outcome, only the NICE guideline (National Institute for Health and Care Excellence, 2015) had an adequate overall quality (over 80% in all domains); this was subsequently used as a content‐related basis for the information leaflet on fall prevention. While designing and creating the layout, the recommendations given in the Plain Language Action Information Network (2011) and Wizowski et al. (2014) were considered. The draft material was discussed with experts and evaluated by nurse practitioners.

Two different information leaflets were created: one for the patients and one for the relatives. The leaflet for relatives listed fewer environmental risk factors, it was more general, and the readers were addressed differently.

### The feedback questionnaire

In order to evaluate the usefulness and understandability of the information leaflets for patients and their relatives, the authors adapted the feedback questionnaire by Wizowski et al. (2014) for fall prevention purposes. The same questionnaire was used for evaluating both information leaflets. The feedback questionnaire contains standardized Likert‐type questions with response options ranging from “strongly disagree (1)” to “strongly agree (5)”. The questions address, for example, if the leaflet is helpful for the reader to prevent falls, easy to understand and if the layout appeals to the reader. Two questions referred to a general assessment, asking the reader if they would recommend the leaflet to others and to describe their overall impression of the leaflet (presentation, content and applicability). The last question could be answered on a five‐point Likert scale, ranging from “very poor (1)” to “very good (5)”.

Furthermore, the feedback questionnaire contained two open‐ended questions. The first open‐ended question gave the participant the opportunity to add further relevant information they felt was missing from the information leaflet, and the second open‐ended question allowed them to make other comments and suggestions. Demographic data on the participants' gender, age and highest completed education were also collected with the feedback questionnaire. The German version of the feedback questionnaire is shown in the supporting information (see Figure S3).

### Statistical analysis

Quantitative data analysis was performed using the statistical programme SPSS version 26 (IBM Corp., 2019) and Microsoft Excel (Microsoft Corporation, 2018). Analysis compared patients' and relatives' answers, and subgroup analyses were performed to investigate questionnaire results with regard to sample characteristics.

In order to analyse the answers to the open‐ended questions on the feedback questionnaire, a qualitative content analysis according to Schreier (2012) was performed. The answers to the open‐ended questions were grouped into four main categories (layout, additional information, understandability and positive feedback). Those main categories were formed by deductive approach and the subcategories by inductive approach. With regard to the formed categories, the aspects are described systematically (Schreier, 2012).

### Ethics committee approval and consent to participate

The feedback questionnaires do not contain any information that might be used to draw conclusions about the individual. All data used were analysed anonymously. The Ethics Committee of the ‘REDACTED’ evaluated the submitted ethics application positively (EC number ‘REDACTED’).

## RESULTS

### Sample characteristics

A total of 125 questionnaires were distributed. Of these, 46 (36.8%) were completed by relatives and 79 (63.2%) were completed by patients. One questionnaire was excluded from the analysis because only the demographic questions were answered. In total, questionnaires from 13 patients had to be excluded because they were not over 65 years old. After data cleaning, 65 (82.28%) of the original 79 patient questionnaires remained. All of the 46 questionnaires from relatives could be used for the analysis. Overall, 111 (88.8%) of the 125 feedback questionnaires were valid.

The sample sizes vary in the analysis, as not every question was answered by the entire sample.

The description of the sample is shown in Table 1. Most of the members of the patients' group were female (53.2%). The most frequently represented patient age group was 71 to 80 (47.7%), and most patients reported compulsory education as their highest completed education (42.6%). More women were also present in the group of relatives (68.9%), and the most frequently reported age group was 51 to 60 years (23.9%). Most relatives had completed an apprenticeship (28.9%).

**TABLE 1:** Characteristics of the included patients and residents.

Sample characteristics	Patients (*n* = 65)	Relatives (*n* = 46)
Sex *n*	62^a^	45^a^
Male *n* (%)	28 (45.2)	14 (31.1)
Female *n* (%)	33 (53.2)	31 (68.9)
Diverse *n* (%)	1 (1.6)	0 (0.0)
Age group *n*	65*	46*
≤21–30 years *n* (%)	‐	5 (10.9)
31–40 years *n* (%)	‐	4 (8.7)
41–50 years *n* (%)	‐	5 (10.9)
51–60 years *n* (%)	‐	11 (23.9)
61–70 years *n* (%)	16 (24.6)	7 (15.2)
71–80 years *n* (%)	31 (47.7)	7 (15.2)
≥81 years *n* (%)	18 (27.7)	7 (15.2)
Highest completed school education *n*	*n* = 61* ^,^ ^a^	*n* = 45* ^,^ ^a^
Compulsory school *n* (%)	26 (42.6)	6 (13.3)
Apprenticeship *n* (%)	13 (21.3)	13 (28.9)
High school diploma *n* (%)	6 (9.8)	8 (17.8)
University *n* (%)	7 (11.5)	8 (17.8)
Other *n* (%)	9 (14.8)	10 (22.2)

^a^ *Different sample sizes, as not all questions were answered by all participants*.

* *Statistically significant difference (p ≤ 0.05)*.

### Patients and relatives

**FIGURE 1:**
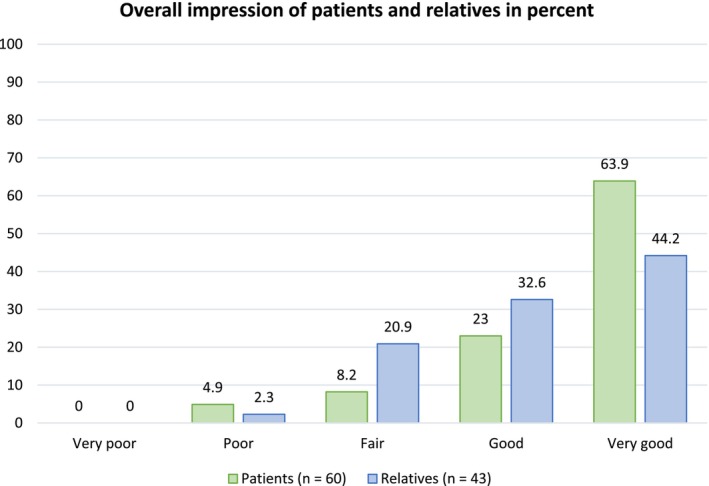
The rating of the overall impression regarding presentation, content and application by patients and relatives in percent. [Colour figure can be viewed at wileyonlinelibrary.com]

The overall question on the impression (presentation, content and applicability) of the information leaflet was found to be very good by the majority of patients (63.9%, *n* = 39), while 44.2% (*n* = 19) of the relatives found the overall impression to be very good and 32.6% (*n* = 14) good. No statistically significant difference was identified between patients and relatives (*p* = 0.059). These results are shown in Figure 1.

More than a half of both patients and relatives strongly agreed that the information leaflet was easy to read and to understand, was helpful and enhanced knowledge on the topic of fall risk, and that the drawings, pictures and pictograms facilitated understanding. Most participants agreed that the information leaflet answered their questions and had an appealing design. The latter was the only one showing a significant difference between patients and relatives (*p* = 0.002), with a slightly lower rating by the participating relatives. The results are shown in Table 2.

**TABLE 2:** Results of the patients' and relatives' analysis.

	Easy to read	Easy to understand	Helpful for me	Knowledge enhancement on the topic of fall risk	Support in the reduction of fall risk	Answering my questions	Recommend to others	Drawings, pictures and pictograms facilitate understanding	Appealing design
Patients *n*	59	60	61	62	62	61	62	60	62
Strongly disagree (%)	5.1	1.7	1.6	3.2	1.6	4.9	1.6	1.6	3.2
Disagree (%)	3.4	3.3	3.3	1.6	3.2	0	1.6	1.6	0
Neither agree nor disagree (%)	8.5	5.0	16.4	14.5	14.5	13.1	8.1	8.1	4.8
Agree (%)	15.3	18.3	21.3	16.1	11.3	18.0	14.5	14.5	21.0
Strongly agree (%)	67.8	71.7	57.4	64.5	69.4	63.9	74.2	74.2	71.0
Relatives *n*	46	46	46	46	46	46	46	46	3.2
Strongly disagree (%)	2.2	2.2	2.2	2.2	4.3	4.3	2.2	2.2	4.3
Disagree (%)	0	0	2.2	4.3	2.2	0	2.2	4.3	6.5
Neither agree nor disagree (%)	6.5	6.5	15.2	13.0	15.2	15.2	17.4	17.4	19.6
Agree (%)	15.2	19.6	17.4	26.1	23.9	26.1	13.0	19.6	23.9
Strongly agree (%)	76.1	71.7	63.0	54.3	54.3	54.3	65.2	56.5	45.7
*p*‐value	0.263	0.948	0.599	0.390	0.175	0.406	0.254	0.312	0.002*

* *Statistically significant difference (p ≤ 0.05)*.

### Subgroup analysis according to gender, age and education

#### Gender

The ratings for the overall impression of the information leaflet were similarly positive in female and male patients, with no significant difference between genders (*p* = 0.921). The majority of female and male patients agreed with all questions (see Table 3).

**TABLE 3:** Results of the overall impression of all subgroups in percent.

Overall impression	Very poor	Poor	Fair	Good	Very good	*p* value
Gender						
Patients *n* = 58						*p* = 0.921
Male *n =* 28 (%)	0	7.1	7.1	25	60.7	
Female *n =* 29 (%)	0	3.4	10.3	20.7	65.5	
Diverse *n = 1* (%)	0	0	0	100	0	
Relatives *n* = 42						*p* = 0.872
Male *n = 14* (%)	0	0	21.4	35.7	42.9	
Female *n = 28* (%)	0	0	21.4	32.1	46.4	
Age group						
Patients *n* = 61						*p* = 0.578
61–70 years *n =* 15 (%)	0	0	6.7	26.7	66.7	
71–80 years *n* = 30 (%)	0	0	13.3	23.3	63.3	
≥81 years *n* = 16 (%)	0	18.8	0	18.8	62.5	
Relatives *n* = 43						*p* = 0.460
21–30 years *n* = 4 (%)	0	25	25	50	0	
31–40 years *n* = 4 (%)	0	0	25	25	50	
41–50 years *n* = 5 (%)	0	0	20	40	40	
51–60 years *n* = 10 (%)	0	0	0	50	50	
61–70 years *n* = 7 (%)	0	0	28.6	28.6	42.9	
71–80 years *n* = 7 (%)	0	0	28.6	0	71.4	
≥81 years *n* = 6 (%)	0	0	33.3	33.3	33.3	
Education groups						
Patients *n* = 58						*p* = 0.940
Compulsory school *n* = 23 (%)	0	4.3	8.7	26.1	60.9	
Apprenticeship *n* = 13 (%)	0	0	7.7	7.7	84.6	
High school diploma *n* = 6 (%)	0	33.3	0	33.3	33.3	
University *n* = 7 (%)	0	0	0	57.1	42.9	
Other *n = 9* (%)	0	0	11.1	11.1	77.8	
Relatives *n* = 42						*p* = 0.486
Compulsory school *n* = 5 (%)	0	0	0	20	80	
Apprenticeship *n* = 12 (%)	0	0	33.3	25	41.7	
High school diploma *n* = 8 (%)	0	0	12.5	62.5	25	
University *n* = 7 (%)	0	14.3	14.3	28.6	42.9	
Other *n =* 10 (%)	0	0	30	20	50	

42.9% (*n* = 6) of male and 46.4% (*n* = 13) of female relatives perceived the overall impression as very good. None of the relatives rated the general impression of the information leaflet as poor. The between‐group difference was not statistically significant (*p* = 0.872) (see Table 3). The majority of the female relatives strongly agreed with all questions. All results are shown in the supporting information (see Table S1).

#### Age

The question regarding the overall impression was rated best by the patients aged 61–70‐years, and 66.7% (*n* = 10) found it very good and 26.7% (*n* = 4) good. Only a few individuals of the age group ≥81 years (18.8%; *n* = 3) rated the overall impression as poor (see Table 3). The majority of all three age groups (61–70, 71–80, ≥ 81) strongly agreed with all questions. The group of the ≥81 years old patients was the one to strongly disagree most frequently, especially regarding if the information leaflet was easy to read (12.5%, *n* = 2), if it answered their questions (17.6%, *n* = 3) and if drawings, pictures or pictograms facilitated understanding (16.7%, *n* = 3). The difference between the age groups was not statistically significant. Both the 61–70 (75%, *n* = 12) and 71–80 (82.1%, *n* = 23) years old patients would recommend the information leaflet to others (see Table S2).

The 51–60‐years old relatives rated the overall impression best (see Table 3). Nobody in the group of the 41–50, 61–70, 71–80 and ≥81 years old relatives disagreed with any of the questions. There was no significant difference in the ratings of the different age subgroups. The group of the 41–50 years old relatives strongly agreed that the information leaflet was helpful and enhanced their knowledge (each 100%; *n* = 5). The vast majority of the age group 51–60 strongly agreed that the information leaflet was easy to read (90.9%; *n* = 10). All results are shown in the supporting information (see Table S2).

#### Educational level

Patients with a university degree rated the overall impression best; 42.9% (*n* = 3) found the information leaflet very good and 57.1% (*n* = 4) good. However, patients who had a high school diploma rated it worst. The difference between the groups was not statistically significant (see Table 3).

The majority of patients of all education groups agreed with all questions. Patients who had completed an apprenticeship strongly agreed that the information leaflet was easy to understand, answered their questions and that they would recommend it to others (each 100%; *n* = 12). There was a statistically significant difference between the education groups regarding if it was easy to read (*p* = 0.06) and easy to understand (*p* = 0.014), with a tendency towards poorer ratings by people with compulsory schooling as their highest level of education. The group differences regarding the other questions were not statistically significant (see Table S3).

The overall impression was rated best by the relatives who had completed compulsory school, 80% (*n* = 4) found it very good and 20% (*n* = 1) good. The difference was not statistically significant. These results are shown in table 2 (see Table 3).

More than a half of all education groups agreed with all questions. The overall rating by relatives who had completed an apprenticeship was the lowest. The group differences regarding the other questions were not statistically significant (see Table S3).

### Results of answers to the open‐ended questions

The coding frame with the codes, definitions, and examples for this analysis is shown in Table 4.

**TABLE 4:** Coding frame for the qualitative content analysis.

	Category	Definition	Example
Main category			
	Layout	Any comment on improving the design of health information and the presentation of results.	
Subcategory			
	Colour scheme	Proposals for Changing the colour design of the health information.	ID 32: more colour
	Writing	Notes on font and/or font size.	ID 32: bigger font
	Type of information	Comments on the way the information is presented.	ID 43: Preparation as a brochure
	Design	Comments on the presentation of health information in terms of pictures, graphics and tables.	ID 31: more pictures
Main category			
	Additional information	Any comments on the content of the health information and the strategies included.	
Subcategory			
	Concrete examples	Notes on further examples of fall prevention.	ID 31: Concrete examples (carpet, movement)
	Activating information	Notes on information to encourage action.	ID 15: Take a walking stick for support
	Information on safety in the home	Comments on further information related to improving safety in the home.	ID 4: Safety in the bathroom shower
	Further tips for preventing falls	Notes on additional advice on how to avoid falls.	ID 4: poorly lit paths
	Information on contact persons	Notes on the professional groups that can help those affected with regard to fall prevention.	ID 16: The experts are not known? Doctors or experts do not feel competent???? Don't have time and so on!!!
	Background information	Notes on general information related to fall.	ID 52: Important for patients is: to know the diagnosis from the doctor!
Main category			
	Understandability	Comments on ambiguities regarding content, presentation of results and/or preparation of information.	ID 31: Why ‘+’ signs? Overall good, just as a suggestion to ‘talk about it’.
Main category			
	Positive feedback	Any nonspecific, positive feedback regarding health information.	ID 46: All in all, very good information!

### Desire for further information

Four patients and three relatives completed the open‐ended statement “I would have liked further information about…”. Two statements made by the patients were related to the category “additional information”. One of the two patients wanted to know more about “safety in the living area”, especially concerning the bathroom and shower area (*“safety in the bathroom‐ shower”*, *patient ID 4*), and the other person wanted to receive more information that would help the reader to take action. One comment made by the relatives was not relevant for the analysis and two referred to the category “additional information”. The statements could still be assigned to individual subcategories. One person wanted more specific examples of how a fall can be avoided: “*Concrete examples on carpets or movement for example (relative*, *ID 31)*.” The other person wanted more information about how to prevent falls as an informal care giver for a person with risk of fall (relative ID 28).

### Other comments and suggestions

The open‐ended question that invited participants to provide “Other comments and suggestions” was answered by a total of twelve people, represented by ten patients and two relatives.

Five statements from the patients could be assigned to the main category “additional information”. Two of these five statements could then be assigned to the subcategory “further advice on fall prevention”, e.g.: *“uneven and poorly lit paths” (patient, ID 4)*, and one statement each could be assigned to the subcategories “activating information” like *“take a walking stick for support” (relative, ID 45)*, “background information” and “information about contact persons” (*“The experts are not known? Doctors or experts do not feel competent???? Do not have time and so on.”*, *patient*, *ID 16*). The last statement referred to the fact that the person did not know exactly who the experts mentioned in the text were. The reader also stated that they felt that these experts would not have time to address such concerns. In the statement assigned to the category “background information”, the person wanted more general information about falls:

Four additional statements were related to the layout of the information leaflet. Comments were made about the font, which was considered to be too small, and to indicate that the use of more colours and more pictures was desirable.

One patient considered the sentences to be too long, and one relative found the “+” symbol used in the information leaflet confusing. Overall, participants responded to the request for “other comments and suggestions” most frequently by providing comments about the layout and questions that could be assigned to the “additional information” category, although no tendency towards a particular category could be identified. The “understanding” and “positive feedback” categories were each represented twice.

## DISCUSSION

In order to find out whether information leaflets meet the needs of the target group, they should be evaluated by the target group (Wizowski et al., 2014). The aim of this work was to evaluate the usefulness and understandability of two information leaflets on fall prevention that were designed for hospital patients and their relatives. The integrated conceptual model of health literacy by Sørensen et al. (2012) shows that the process of health literacy requires the ability to understand the health information, the ability to interpret, filter, judge and evaluate the health information, and the ability to communicate and use the information (Sørensen et al., 2012).

The vast majority of the patients and relatives found the information leaflets useful and understandable. Only one question showed a statistically significant difference between the groups of patients and relatives. The majority of patients and relatives fully agreed that they would recommend the information leaflets to others, and the majority of patients and relatives rated the overall question with “very good” and/or “good”. These results are very satisfactory, as they show that the information leaflets were already well‐tailored to meet the needs of the target group, in contrast to the results of other studies (Posch et al., 2020). It is important to offer well‐tailored information leaflets because most people trust in information from health care professionals. However, especially individuals with limited health literacy tend to use and trust more often sources such as social media and blogs. Those sources might contain low‐quality health information. It is therefore of critical importance to also reach this audience with high‐quality information (Chen et al., 2018).

Information leaflets should be created using the concept of Plain Language so that they are as understandable as possible (Plain Language Action Information Network, 2011; Wizowski et al., 2014). Participants made a few comments regarding the understandability. For example, participants wanted sentences to be shorter, fonts to be larger, or wanted to have more information about experts who could assist them in fall prevention. No comments were made on the language used or the choice of words; however, one statement related to the meaning of the symbol “+” was made. The information leaflets for patients and relatives contains a legend on the first page describing this symbol. This was apparently overlooked, possibly because the font size was too small.

The layout seems to be particularly important for users of information leaflets. In the study by Dellson et al. (2016), where information leaflets were evaluated in focus group interviews, a high proportion of the feedback was related to the layout. In the evaluation of our information leaflets, patients commented on the layout particularly often. More colours and more pictures were desired. Images in information leaflets can improve the patients' ability to understand the material (Schubbe et al., 2020). However, according to Houts et al. (2006) and Lühnen et al. (2018), no clear recommendation can be made regarding the use of images, as each person reacts differently to images. However, recent studies show that the use of pictograms made it significantly easier for readers to understand the content of an information leaflet (Dowse, 2021; Sekhar et al., 2017). The evaluated information leaflets contained pictograms that functioned as bullet points and reflected the content of each statement. On average, patients and relatives were more likely to agree that the pictograms in these information leaflets helped them to understand the content.

Furthermore, a larger font was desired. At least a 12‐pt font size is the recommended size in the literature (Griffin et al., 2003; Steckelberg et al., 2005). The investigated information leaflets used a 12‐pt font size. The length of sentences is important for understandability. Short sentences are best (Coulter et al., 2006). One participant would have liked shorter sentences. On average, both patients and relatives tended to agree or fully agreed that the layout was appealing.

Although most of the content of the information leaflets was found to be useful, some comments were made that could further increase its usefulness. For an information leaflet to be useful for readers, it must contain specific instructions for action. These must also be practicable for the target group (Kessels, 2003; Wizowski et al., 2014). Both patients and relatives most frequently wanted more additional information. For example, they desired more tips on how to avoid falls or specific examples or information about safety in the living area. The patients would have liked more activating information and more tips on fall prevention. The relatives wanted more concrete examples and further advice on fall prevention. The study by Schmitz et al. (2010) also indicated that many people would have expected more concrete instructions for action.

Different information leaflets were prepared for patients and relatives. The biggest difference was that the information leaflet for relatives listed fewer environmental risk factors, such as carpets and lighting. In the information leaflet for relatives, this part contained more general information. The content analysis results also show that relatives desire more precise information about fall prevention measures. According to Kessels (2003), specific instructions in an information leaflet are better than general statements. Information that is more specific was also requested about the “experts”. A multitude of responsible parties can act to prevent falls. Therefore, it is hard for a layperson to identify the right experts, and the respective experts who play a role in fall prevention should be clearly defined. Especially older adults have a strong need to be in control of their own health and well‐being. However, they need the help of health care professionals and the opportunity to interact with them (de Wit et al., 2020).

One patient noted that they would have liked to have more information on the topic of safety in the bathroom and shower. This point is briefly mentioned in the health information for patients. According to studies, falls occur very frequently in the bathroom, and they can have especially serious consequences there (Keglovits et al., 2020; Kumfo, 2017; Neslihan & Belgin, 2013). Therefore, more information and assistance in this area should be provided. Based on the results of the evaluation, the information leaflets were adapted. The adapted leaflets and a table with the changes made can be found in the Supporting Information (Figures S1 and S2, Table S4).

According to the study by Tille et al. (2019), difficulty in understanding written information leaflets correlates strongly with basic education and older age. Individuals with an intermediate or basic education and those over the age of 65 have a harder time understanding this information (Tille et al., 2019). The study by Jindal and MacDermid (2017) also showed that the readers' education influences their understanding of information leaflets. Our results show that, in this evaluation, a statistically significant difference was only observed between education levels among patients regarding understandability. However, a statistically significant difference was identified among patients' educational groups regarding the questions “easy to read” and “easy to understand”. These results are quite similar to the results of the study by Lins et al. (2011). This study of an information leaflet for individuals over the age of 60 showed no statistically significant difference between education levels (Lins et al., 2011).

The majority of both male and female patients and relatives rated all questions as good. The patients' age group ≥81 years and the relatives' age group 51–60 years rated all questions the worst.

### Methodological limitations

The sample was small, and many questions were not answered. Especially the open‐ended questions were answered only in few cases, which limits the validity of the study. Since the survey was conducted during the first COVID‐19 (coronavirus disease 2019) pandemic wave, the desirable number of 180 participants could not be achieved. In the end, 111 of 125 feedback questionnaires were considered as valid. Relatives were only allowed to be present on the wards for a limited number and time (one person, 15 min per day), which could be a possible explanation for the low number of participating relatives. It is possible that relatives preferred to talk with their family members during this limited time rather than complete feedback questionnaires. Furthermore, nurses had heavy demands on their time and resources during this time, which was potentially why no additional effort was made to increase the sample size. The feedback questionnaire was standardized for patients and relatives, and so were the response categories for the age groups, which were divided in ten‐year increments. Therefore, it cannot be determined in retrospect whether all patients who had ticked the age group 61–70 years were actually over 65 years old.

Even if there is a potential for bias and information loss, there is a strong support for an assumption that the information loss is minimal in Likert‐scaled surveys (Westland, 2022).

## CONCLUSION

The evaluation of the information leaflets on fall prevention for patients and relatives using a feedback questionnaire shows that, overall, the developed leaflets are useful and understandable for the target group. Only a few adaptations were recommended. The critical comments were often made by single individuals; nevertheless, these can contribute to the improvement of the information leaflets. The evaluation has demonstrated the importance of providing clear tips and recommendations to patients and relatives, so that the target group knows exactly what they can do to prevent falls.

A further evaluation regarding the benefit of the fall prevention leaflets in discharge management should be performed. This may include an examination of how to improve knowledge, implemented changes in the home environment or record fall incidences. Furthermore, the leaflet must be updated regularly to ensure that it remains complete and up to date.

## CONFLICT OF INTEREST STATEMENT

No conflict of interest.

## Supporting information


**Data S1:** Supporting Information
