# Supplementary material for: Evaluation of the usefulness and understandability of information leaflets on fall prevention from the perspective of hospital patients and their relatives
Source: Health Info Libr J. 2024 Apr 30;42(1):83–95. doi: 10.1111/hir.12531 (PMC12590335; doi:10.1111/hir.12531)
Supplement: Supplementary file 1 — Data S1: Supporting Information [file HIR-42-83-s001.docx]

**Supporting Information, Figure 1:** Adapted information leaflet on fall prevention for patients

**Patient Information**

**How to prevent falls at your home**

**Dear Patients,**

There are numerous causes of falls. This information leaflet describes measures you can take to prevent falls at your home.

Attach patient sticker here or fill in data by hand

Patient name:

Date of birth:

The risk of falls among both men and women **increases with advancing age**. One in three persons over 65 years of age experiences a fall incident at least once a year. Possible causes of falls may be a **changed gait pattern, medication intake, or certain health conditions**.

A fall and its consequences pose a considerable burden for the affected individual as well as for their relatives and friends. Therefore, it is very important to take measures to prevent falls.

You can take the following measures in your home to reduce the risk of falling or to prevent falls.

**Many falls can be prevented by making some minor changes in your daily life!**

# **Am I at a higher risk of falling?**

If one of the following is true for you, then you are at an increased risk of falling:

-
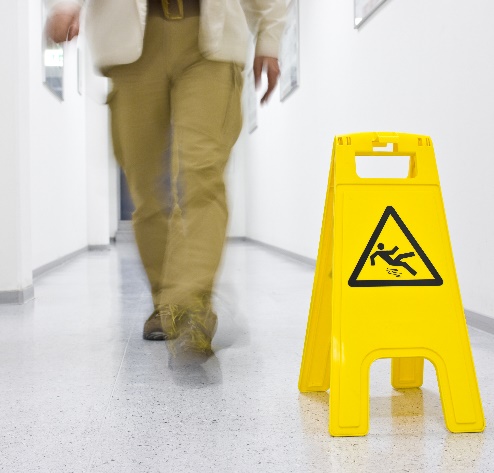
Fall incidents within the previous 6 months
- A changed **gait pattern** (e.g. unsteadiness on your feet or insecurity when walking) **or diminished balance**
- Intake of certain **medication** (e.g. sleeping tablets or sedatives)
- A health condition affecting your **memory** (e.g. dementia)
- A **bladder condition or incontinence problems**

©alterfalter/Shutterstock.com

- **Dizziness/light-headedness** and **fainting**
- Impaired **vision**
- Inappropriate footwear without heel support/fixation (e.g. slippers)

# **What are the possible consequences of a fall?**

A fall may cause **injuries** and **bone fractures**. Fractured wrists and fractures of the femoral neck, the hip, or the torso bones are the most common consequences of a fall event. Osteoporosis (bone loss, brittle bones) further increases the risk of bone fracture after a fall. Women are markedly more often affected by fractures caused by falls. Fall-related injuries may result in hospital admission.

Persons who have experienced a fall often develop a **fear** of falling again and become insecure in their daily lives. They may lose confidence in their capability to perform certain tasks and activities without the help of others. This may have an adverse effect on their **mobility** and their quality of life.

# **Who can assess my fall risk?**

We suggest you to consult with experts on a regular basis to assess your **personal fall risk**, i.e. general practitioners, nurses, or physiotherapists. These experts evaluate and assess your **gait and balance status**, amongst other factors. Some **medications** may also increase the risk of falling or cause falls. Discuss this aspect of your medication regime with your general practitioner.

Have your vision checked regularly by an ophthalmologist or optician.

If you feel discomfort or pain in your feet or have questions regarding your footwear, consult with a podiatry specialist, orthopaedic physician, podiatrist, or orthopaedic technician.

**It is impossible to eliminate every fall risk.
However, you can address some fall risks and thus prevent falls.**

# **How can I reduce fall risks in my home environment?**


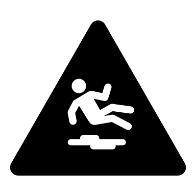
**Check your home environment for fall risks and trip hazards!** For this purpose, you may also use the safety checklist for fall prevention in the home environment (see attachment).

- Clear the floor from obstacles and trip hazards like mats, carpets, or cords. Have any damaged stairs or flooring repaired. Use non-slip bath mats.


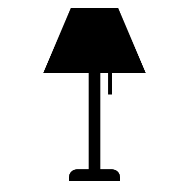


- Ensure adequate lighting at all times of the day. For instance, install a night light in your hallway and attach glow-in-the-dark-stickers to stairs and objects that may be trip hazards in the dark.
-
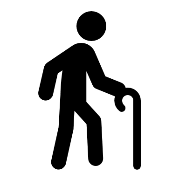
Pay attention to have your aids and devices (e.g. cane, walking aid, cell phone, medical alert system) within reach at all times. Install grab bars and handrails if and where needed – and do use them! Get a raised toilet seat to make getting up easier.


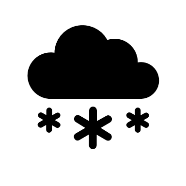


- Outside, avoid hazardous (uneven) walkways. In winter/icy weather, only use walkways that have been cleared of ice or use traction aides. Get help for your outdoor winter work (shovelling snow, dispersing sand/salt).

**What can I do to minimize my fall risk?**

**Pay attention to adequate clothing and proper footwear!**

-
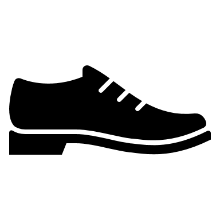
It is important that you always wear **solid shoes**! Your footwear should fit properly and support you well. Shoes should have non-slip soles and heels that are not too high, so you can walk around safely and steadily. You should also wear well-fitting shoes at your home (no slippers!).
-
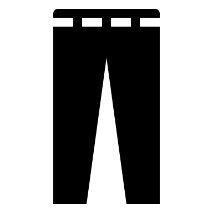
Make sure your clothes are the right size. If your **trousers/skirts/night gowns are too long, have them shortened**. Your clothes should never touch the floor when either walking or sitting.

**Seek/accept help!**

-
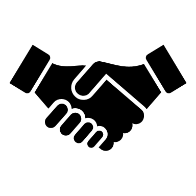
**Delegate** certain tasks in and around the house! For example, **get help** if you need to hang up new curtains, trim hedges, or do any jobs that involve climbing a ladder. Your relatives and friends will certainly be willing to support you with strenuous tasks or ones that require you to perform work above the head.


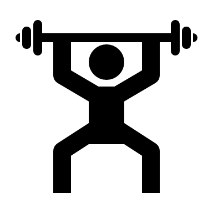
**Take measures to promote your physical fitness!**

- **Regular strength and balance exercises** may reduce falls and fall-related injuries. Especially women profit from regular exercise to effectively reduce falls and fractures.
- **
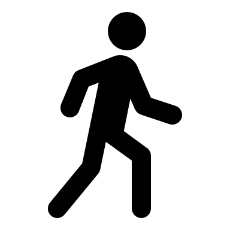
Any kind of physical activity** (e.g. taking walks, gymnastics exercises) is good for your physical fitness. However, if your ability to walk or your physical strength is restricted, then you need specific exercises that are tailored to your personal needs. **Consult a physiotherapist or occupational therapist** for such suitable exercises.


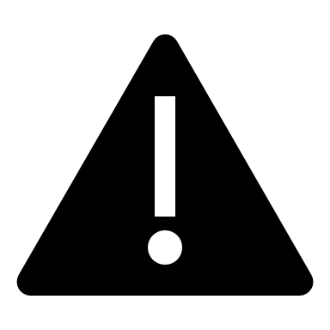


- After a fall, take care to discuss the incident with your general practitioner or a nurse. They may help you to identify the possible causes of your fall incident. This enables you to plan measures in order to prevent future falls.
- **Talk with your relatives and friends** – discuss **the topic of fall risk** openly with them and exchange ideas about how you might make your living environment a safer place for you.
- **Accept support** and offered help – both from health experts (general practitioners, nurses, therapists) and from your relatives and friends.

**If anything is unclear or questions arise**, please contact your general practitioner or a nurse (e.g. from a home care service provider).

We are grateful to the 'REDACTED' for their support in creating this leaflet.

**Masthead:** Media proprietor: 'REDACTED'
For questions: 'REDACTED'

**Supporting Information, Figure 2:** Adapted **i**nformation leaflet on fall prevention for relatives

**Information for relatives and significant others**

**How to help prevent falls at home**

Attach patient sticker here or fill in data by hand

Patient name:

Date of birth:

**Dear Relatives and Friends,**

There are numerous causes of falls. This information leaflet informs about falls and fall risks and describes measures you can take to help prevent falls at home.

The risk of falls among both men and women **increases with advancing age**. One in three persons over 65 years of age experiences a fall incident at least once a year. Possible causes of falls may be a **changed gait pattern, medication intake, or certain health conditions**.

A fall and its consequences pose a considerable burden, not only for the affected individual but also for their relatives and friends. Therefore, it is very important to take measures to prevent falls.

**Many falls can be prevented by making some minor changes in one’s daily life!**

**When is your relative/friend at a higher risk of falling?**

If one of the following is true for your relative/friend, then they are at an increased risk of falling.

- **
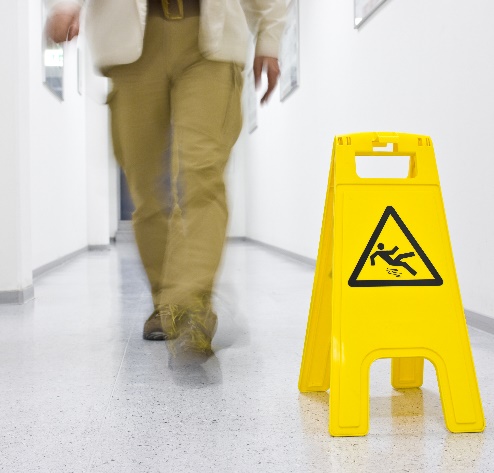
Fall incidents** within the previous 6 months
- A changed **gait pattern** (e.g. unsteadiness on their feet or insecurity when walking) **or diminished balance**
- Intake of certain **medication** (e.g. sleeping tablets or sedatives)

@ alterfalter/Shutterstock.com

- A health condition affecting **memory** (e.g. dementia)
- A **bladder condition or incontinence problems**
- **Dizziness/light-headedness** and **fainting**
- Impaired vision

**Not all of these circumstances can be alleviated. However, a few risks may be reduced by making some minor adjustments or behavioural changes.**

**What are the possible consequences of a fall?**

A fall may cause **injuries** and **bone fractures**. Fractured wrists and fractures of the femoral neck, the hip, or the torso bones are the most common consequences of a fall event. Osteoporosis (bone loss, brittle bones) further increases the risk of bone fracture after a fall. Women are affected much more often by fractures caused by falls. Fall-related injuries may result in the hospital admission of your relative/friend.

Persons who have experienced a fall often develop a **fear** of falling again and become insecure in their daily lives. They may lose confidence in their capability to perform certain tasks and activities without the help of others. This may have an adverse effect on their **mobility** and their quality of life.

**Who can assess somebody’s fall risk?**

A person’s **individual fall risk** should be assessed on a regular basis by health experts, i.e. general practitioners, nurses, or physiotherapists. These experts evaluate and assess the **gait and balance status** of your relative/friend, amongst others. If requested, a general practitioner may also examine the person’s **medication** regime with respect to increasing fall risk or contributing to falls.

**You can help to reduce fall risks and to prevent fall incidents!**

**How can I support my relative/friend?**

**Motivation is an important factor in helping people to actually carry out planned changes in their daily lives.**

-
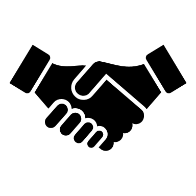
Motivate your relative/friend to **make their home environment a safer place**. Set up a plan together with possible changes. It is best to start in one room. Remove possible trip hazards (e.g. mats, carpets, cords). If needed, install handrails, grab bars, raised toilet seats, or non-slip bath mats. Make sure the areas are well-lit at all times of the day.


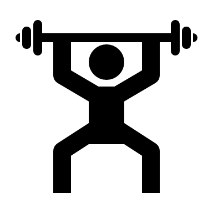


- Motivate your relative/friend to be **physically active**. For instance, take walks together. Regular activity reduces a person’s fall risk.
-
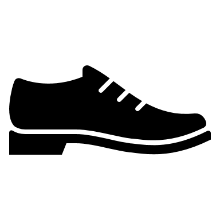
Is your relative/friend restricted in their ability to walk or is their balance poor? Consult a professional health expert! Physiotherapists can select and compile suitable **strength and balance exercises** that are tailored to meet your relative’s/friend’s individual needs. Especially women profit from regular exercise to effectively reduce falls and fractures.

In addition, podiatry specialists, orthopaedic physicians, podiatrists, or orthopaedic technicians may help with foot or footwear problems.

- Motivate your relative/friend to have their vision checked regularly and to wear their visual aids.
-
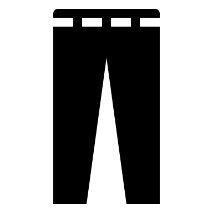
Recommend your relative/friend to only wear **clothes that are the right size** and to have too long trousers/skirts/nightgowns shortened. Clothes should never touch the floor when either walking or sitting.
-
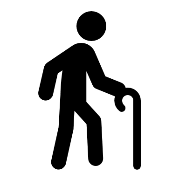
Certain tasks in and around the house increase the risk of falling (e.g. tasks requiring one to perform work above the head, like hanging up curtains, or to carry out tasks on uneven/slippery surfaces). Encourage your relative/friend to **accept help** and offer active support.

- Use the **safety checklist** for fall prevention in the home environment (see attachment).


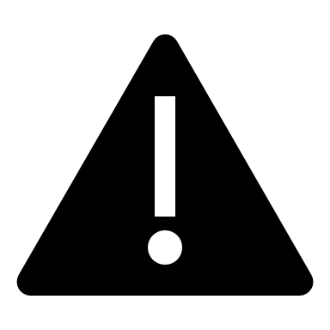
**Important!**

**If there has been a fall incident, discuss it openly with your friend/relative**. Together with a general practitioner or nurse, the possible causes of the fall can be identified to plan measures in order to prevent future falls.

**If anything is unclear or questions arise**, please contact a general practitioner or a nurse (e.g. from a home care service provider).

We are grateful to the 'REDACTED' for their support in creating this leaflet. **Masthead:** Media proprietor: 'REDACTED'
For questions: 'REDACTED'


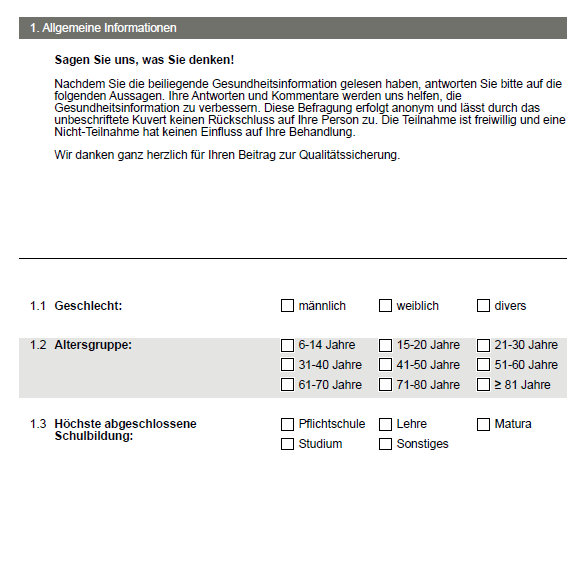
**Supporting Information, Figure 3:** German version of the feedback questionnaire


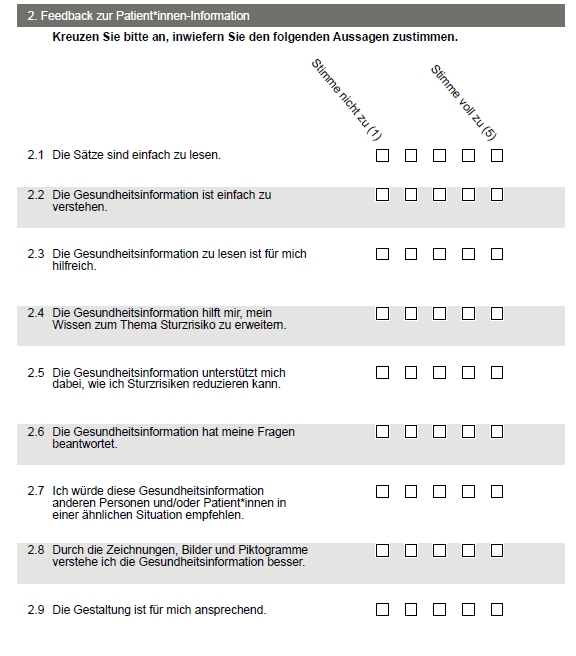


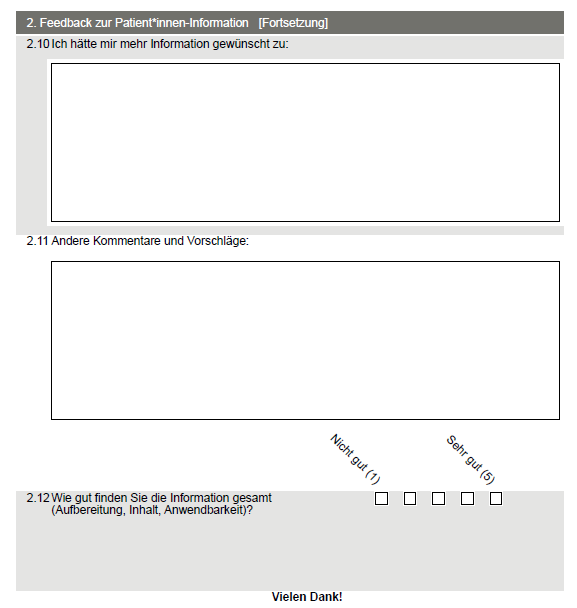


**Supporting Information, Table 1:** Results of the subgroup analysis according to gender

| **Gender** |  | **Easy to read** | **Easy to understand** | **Helpful for me** | **Knowledge enhancement on the topic of fall risk** | **Support in the reduction of fall risk** | **Answering my questions** | **Recommend to others** | **Drawings, pictures and pictograms facilitate understanding** | **Appealing design** |
| --- | --- | --- | --- | --- | --- | --- | --- | --- | --- | --- |
| **Patients** |  |  |  |  |  |  |  |  |  |  |
| Male *n* |  | 26 | 26 | 25 | 27 | 26 | 25 | 26 | 25 | 26 |
|  | Strongly disagree (%) | 7.7 | 3.8 | 4 | 3.7 | 3.8 | 4 | 3.8 | 4 | 3.8 |
|  | Disagree (%) | 0 | 0 | 0 | 0 | 3.8 | 0 | 0 | 4 | 0 |
|  | Neither agree nor disagree (%) | 3.8 | 3.8 | 16 | 14.8 | 11.5 | 12 | 7.7 | 16 | 3.8 |
|  | Agree (%) | 19.2 | 19.2 | 32 | 11.1 | 7.7 | 28 | 15.4 | 16 | 34.6 |
|  | Strongly agree (%) | 69.2 | 73.1 | 48 | 70.4 | 73.1 | 56 | 73.1 | 60 | 57.7 |
| Female *n* |  | 30 | 30 | 32 | 31 | 32 | 32 | 32 | 31 | 32 |
|  | Strongly disagree (%) | 3.3 | 0 | 0 | 3.2 | 0 | 6.3 | 0 | 6.5 | 3.1 |
|  | Disagree (%) | 6.7 | 6.7 | 6.3 | 3.2 | 3.1 | 0 | 3.1 | 3.2 | 0 |
|  | Neither agree nor disagree (%) | 13.3 | 6.7 | 18.8 | 16.1 | 18.8 | 12.5 | 6.3 | 9.7 | 6.3 |
|  | Agree (%) | 10 | 16.7 | 15.6 | 19.4 | 15.6 | 12.5 | 15.6 | 6.5 | 9.4 |
|  | Strongly agree (%) | 66.7 | 70 | 59.4 | 58.1 | 62.5 | 68.8 | 75 | 74.2 | 81.3 |
| Diverse *n* |  | 1 | 1 | 1 | 1 | 1 | 1 | 1 | 1 | 1 |
|  | Strongly disagree (%) | 0 | 0 | 0 | 0 | 0 | 0 | 0 | 0 | 0 |
|  | Disagree (%) | 0 | 0 | 0 | 0 | 0 | 0 | 0 | 0 | 0 |
|  | Neither agree nor disagree (%) | 0 | 0 | 0 | 0 | 0 | 100 | 100 | 0 | 0 |
|  | Agree (%) | 100 | 100 | 0 | 100 | 0 | 0 | 0 | 100 | 100 |
|  | Strongly agree (%) | 0 | 0 | 100 | 0 | 100 | 0 | 0 | 0 | 0 |
| ***p* - value** |  | 0.524 | 0.509 | 0.493 | 0.270 | 0.655 | 0.794 | 0.785 | 0.531 | 0.201 |
| **Relatives** |  |  |  |  |  |  |  |  |  |  |
| Male *n* |  | 14 | 14 | 14 | 14 | 14 | 14 | 14 | 14 | 14 |
|  | Strongly disagree (%) | 0 | 0 | 0 | 0 | 0 | 0 | 0 | 0 | 0 |
|  | Disagree (%) | 0 | 0 | 0 | 7.1 | 0 | 0 | 0 | 0 | 7.1 |
|  | Neither agree nor disagree (%) | 7.1 | 14.3 | 28.6 | 21.4 | 21.4 | 21.4 | 28.6 | 21.4 | 14.3 |
|  | Agree (%) | 21.4 | 14.3 | 21.4 | 14.3 | 35.7 | 28.6 | 7.1 | 21.4 | 42.9 |
|  | Strongly agree (%) | 71.4 | 71.4 | 50 | 57.1 | 42.9 | 50 | 64.3 | 57.1 | 35.7 |
| Female *n* |  | 31 | 31 | 31 | 31 | 31 | 31 | 31 | 31 | 31 |
|  | Strongly disagree (%) | 3.2 | 3.2 | 3.2 | 3.2 | 3.2 | 3.2 | 3.2 | 3.2 | 3.2 |
|  | Disagree (%) | 0 | 0 | 3.2 | 3.2 | 3.2 | 0 | 0 | 3.2 | 6.5 |
|  | Neither agree nor disagree (%) | 6.5 | 3.2 | 6.5 | 6.5 | 12.9 | 12.9 | 12.9 | 16.1 | 22.6 |
|  | Agree (%) | 12.9 | 19.4 | 16.1 | 32.3 | 19.4 | 25.8 | 16.1 | 19.4 | 16.1 |
|  | Strongly agree (%) | 77.4 | 74.2 | 71 | 54.8 | 61.3 | 58.1 | 67.7 | 58.1 | 51.6 |
| ***p* – value** |  | 0.744 | 0.780 | 0.199 | 0.824 | 0.385 | 0.621 | 0.709 | 0.043 | 0.740 |
|  |  |  |  |  |  |  |  |  |  |  |

**Supporting Information, Table 2:** Results of the subgroup analysis according to age

| **Age group** |  | **Easy to read** | **Easy to understand** | **Helpful for me** | **Knowledge enhancement on the topic of fall risk** | **Support in the reduction of fall risk** | **Answering my questions** | **Recommend to others** | **Drawings, pictures and pictograms facilitate understanding** | **Appealing design** |
| --- | --- | --- | --- | --- | --- | --- | --- | --- | --- | --- |
| **Patients** |  |  |  |  |  |  |  |  |  |  |
| 61-70 years *n* |  | 16 | 15 | 15 | 16 | 16 | 15 | 16 | 14 | 16 |
|  | Strongly disagree (%) | 6.3 | 0 | 0 | 0 | 0 | 0 | 0 | 0 | 0 |
|  | Disagree (%) | 6.3 | 6.7 | 6.7 | 0 | 0 | 0 | 0 | 7.1 | 0 |
|  | Neither agree nor disagree (%) | 6.3 | 6.7 | 20 | 25 | 25 | 20 | 25 | 0 | 12.5 |
|  | Agree (%) | 18.8 | 13.3 | 6.7 | 12.5 | 6.3 | 13.3 | 0 | 21.4 | 25 |
|  | Strongly agree (%) | 62.5 | 73.3 | 66.7 | 62.5 | 68.8 | 66.7 | 75 | 71.4 | 62.5 |
| 71-80 years *n* |  | 27 | 29 | 29 | 29 | 28 | 29 | 28 | 28 | 28 |
|  | Strongly disagree (%) | 0 | 0 | 0 | 0 | 0 | 0 | 0 | 0 | 0 |
|  | Disagree (%) | 0 | 3.4 | 0 | 0 | 3.6 | 0 | 0 | 3.6 | 0 |
|  | Neither agree nor disagree (%) | 7.4 | 3.4 | 20.7 | 13.8 | 14.3 | 6.9 | 0 | 10.7 | 3.6 |
|  | Agree (%) | 14.8 | 20.7 | 31 | 13.8 | 10.7 | 24.1 | 17.9 | 14.3 | 25 |
|  | Strongly agree (%) | 77.8 | 72.4 | 48.3 | 72.4 | 71.4 | 69 | 82.1 | 71.4 | 71.4 |
| ≥ 81 years *n* |  | 16 | 16 | 17 | 17 | 18 | 17 | 18 | 18 | 18 |
|  | Strongly disagree (%) | 12.5 | 6.3 | 5.9 | 11.8 | 5.6 | 17.6 | 5.6 | 16.7 | 11.1 |
|  | Disagree (%) | 6.3 | 0 | 5.9 | 5.9 | 5.6 | 0 | 5.6 | 0 | 0 |
|  | Neither agree nor disagree (%) | 12.5 | 6.3 | 5.9 | 5.9 | 5.6 | 17.6 | 5.6 | 22.2 | 0 |
|  | Agree (%) | 12.5 | 18.8 | 17.6 | 23.5 | 16.7 | 11.8 | 22.2 | 0 | 11.1 |
|  | Strongly agree (%) | 56.3 | 68.8 | 64.7 | 52.9 | 66.7 | 52.9 | 61.1 | 61.1 | 77.8 |
| ***p* – value** |  | 0.630 | 0.800 | 0.921 | 0.546 | 0.900 | 0.299 | 0.454 | 0.269 | 0.414 |
| **Relatives** |  |  |  |  |  |  |  |  |  |  |
| 21-30 years *n* |  | 5 | 5 | 5 | 5 | 5 | 5 | 5 | 5 | 5 |
|  | Strongly disagree (%) | 0 | 0 | 0 | 0 | 20 | 20 | 0 | 0 | 20 |
|  | Disagree (%) | 0 | 0 | 0 | 0 | 0 | 0 | 20 | 40 | 40 |
|  | Neither agree nor disagree (%) | 0 | 20 | 60 | 40 | 40 | 40 | 20 | 20 | 20 |
|  | Agree (%) | 20 | 20 | 0 | 40 | 0 | 20 | 0 | 20 | 20 |
|  | Strongly agree (%) | 80 | 60 | 40 | 20 | 40 | 20 | 60 | 20 | 0 |
| 31-40 years *n* |  | 4 | 4 | 4 | 4 | 4 | 4 | 4 | 4 | 4 |
|  | Strongly disagree (%) | 0 | 0 | 0 | 0 | 0 | 0 | 0 | 0 | 0 |
|  | Disagree (%) | 0 | 0 | 0 | 25 | 0 | 0 | 0 | 0 | 25 |
|  | Neither agree nor disagree (%) | 0 | 0 | 25 | 0 | 25 | 25 | 25 | 25 | 0 |
|  | Agree (%) | 25 | 25 | 0 | 25 | 0 | 25 | 0 | 0 | 0 |
|  | Strongly agree (%) | 75 | 75 | 75 | 50 | 75 | 50 | 75 | 75 | 75 |
| 41-50 years *n* |  | 5 | 5 | 5 | 5 | 5 | 5 | 5 | 5 | 5 |
|  | Strongly disagree (%) | 0 | 0 | 0 | 0 | 0 | 0 | 0 | 0 | 0 |
|  | Disagree (%) | 0 | 0 | 0 | 0 | 0 | 0 | 0 | 0 | 0 |
|  | Neither agree nor disagree (%) | 20 | 0 | 0 | 0 | 0 | 0 | 0 | 0 | 0 |
|  | Agree (%) | 0 | 20 | 0 | 0 | 20 | 0 | 20 | 20 | 20 |
|  | Strongly agree (%) | 80 | 80 | 100 | 100 | 80 | 100 | 80 | 80 | 80 |
| 51-60 years *n* |  | 11 | 11 | 11 | 11 | 11 | 11 | 11 | 11 | 11 |
|  | Strongly disagree (%) | 9.1 | 9.1 | 9.1 | 9.1 | 9.1 | 9.1 | 9.1 | 9.1 | 9.1 |
|  | Disagree (%) | 0 | 0 | 9.1 | 9.1 | 9.1 | 0 | 0 | 0 | 0 |
|  | Neither agree nor disagree (%) | 0 | 0 | 0 | 9.1 | 9.1 | 18.2 | 9.1 | 9.1 | 9.1 |
|  | Agree (%) | 0 | 18.2 | 9.1 | 9.1 | 9.1 | 9.1 | 18.2 | 18.2 | 27.3 |
|  | Strongly agree (%) | 90.9 | 72.7 | 72.7 | 63.6 | 63.6 | 63.6 | 63.6 | 63.6 | 54.5 |
| 61-70 years *n* |  | 7 | 7 | 7 | 7 | 7 | 7 | 7 | 7 | 7 |
|  | Strongly disagree (%) | 0 | 0 | 0 | 0 | 0 | 0 | 0 | 0 | 0 |
|  | Disagree (%) | 0 | 0 | 0 | 0 | 0 | 0 | 0 | 0 | 0 |
|  | Neither agree nor disagree (%) | 0 | 0 | 14.3 | 14.3 | 0 | 0 | 14.3 | 28.6 | 28.6 |
|  | Agree (%) | 28.6 | 28.6 | 14.3 | 57.1 | 71.4 | 57.1 | 28.6 | 28.6 | 57.1 |
|  | Strongly agree (%) | 71.4 | 71.4 | 71.4 | 28.6 | 28.6 | 42.9 | 57.1 | 42.9 | 14.3 |
| 71-80 years *n* |  | 7 | 7 | 7 | 7 | 7 | 7 | 7 | 7 | 7 |
|  | Strongly disagree (%) | 0 | 0 | 0 | 0 | 0 | 0 | 0 | 0 | 0 |
|  | Disagree (%) | 0 | 0 | 0 | 0 | 0 | 0 | 0 | 0 | 0 |
|  | Neither agree nor disagree (%) | 0 | 0 | 0 | 0 | 14.3 | 0 | 28.6 | 14.3 | 28.6 |
|  | Agree (%) | 14.3 | 14.3 | 28.6 | 28.6 | 14.3 | 28.6 | 0 | 14.3 | 0 |
|  | Strongly agree (%) | 85.7 | 85.7 | 71.4 | 71.4 | 71.4 | 71.4 | 71.4 | 71.4 | 71.4 |
| ≥ 81 years *n* |  | 7 | 7 | 7 | 7 | 7 | 7 | 7 | 7 | 7 |
|  | Strongly disagree (%) | 0 | 0 | 0 | 0 | 0 | 0 | 0 | 0 | 0 |
|  | Disagree (%) | 0 | 0 | 0 | 0 | 0 | 0 | 0 | 0 | 0 |
|  | Neither agree nor disagree (%) | 28.6 | 28.6 | 28.6 | 28.6 | 28.6 | 28.6 | 28.6 | 28.6 | 42.9 |
|  | Agree (%) | 28.6 | 14.3 | 57.1 | 28.6 | 42.9 | 42.9 | 14.3 | 28.6 | 28.6 |
|  | Strongly agree (%) | 42.9 | 57.1 | 14.3 | 42.9 | 28.6 | 28.6 | 57.1 | 42.9 | 28.6 |
| ***p* – value** |  | 0.215 | 0.894 | 0.355 | 0.831 | 0.696 | 0.877 | 0.797 | 0.772 | 0.791 |

**Supporting Information, Table 3:** Results of the subgroup analysis according to education

| **Education groups** |  | **Easy to read** | **Easy to understand** | **Helpful for me** | **Knowledge enhancement on the topic of fall risk** | **Support in the reduction of fall risk** | **Answering my questions** | **Recommend to others** | **Drawings, pictures and pictograms facilitate understanding** | **Appealing design** |
| --- | --- | --- | --- | --- | --- | --- | --- | --- | --- | --- |
| **Patients** |  |  |  |  |  |  |  |  |  |  |
| Compulsory school *n* |  | 25 | 24 | 25 | 24 | 25 | 26 | 25 | 24 | 25 |
|  | Strongly disagree (%) | 0 | 0 | 0 | 0 | 0 | 7.7 | 0 | 0 | 0 |
|  | Disagree (%) | 8 | 8.3 | 8 | 4.2 | 4 | 0 | 4 | 4.2 | 0 |
|  | Neither agree nor disagree (%) | 20 | 12.5 | 16 | 12.5 | 20 | 19.2 | 12 | 12.5 | 8 |
|  | Agree (%) | 20 | 29.2 | 24 | 29.2 | 16 | 19.2 | 20 | 8.3 | 16 |
|  | Strongly agree (%) | 54 | 50 | 52 | 54.2 | 60 | 53.8 | 64 | 75 | 76 |
| Apprenticeship *n* |  | 11 | 12 | 12 | 12 | 12 | 12 | 12 | 12 | 12 |
|  | Strongly disagree (%) | 9.1 | 0 | 0 | 8.3 | 0 | 0 | 0 | 8.3 | 8.3 |
|  | Disagree (%) | 0 | 0 | 0 | 0 | 0 | 0 | 0 | 0 | 0 |
|  | Neither agree nor disagree (%) | 0 | 0 | 16.7 | 8.3 | 8.3 | 0 | 0 | 8.3 | 0 |
|  | Agree (%) | 0 | 0 | 8.3 | 0 | 0 | 0 | 0 | 8.3 | 8.3 |
|  | Strongly agree (%) | 90.9 | 100 | 75 | 83.3 | 91.7 | 100 | 100 | 75 | 83.3 |
| High school diploma *n* |  | 6 | 6 | 6 | 6 | 6 | 6 | 6 | 6 | 6 |
|  | Strongly disagree (%) | 16.7 | 16.7 | 16.7 | 16.7 | 16.7 | 16.7 | 16.7 | 33.3 | 16.7 |
|  | Disagree (%) | 0 | 0 | 0 | 0 | 0 | 0 | 0 | 0 | 0 |
|  | Neither agree nor disagree (%) | 0 | 0 | 16.7 | 16.7 | 33.3 | 16.7 | 16.7 | 33.3 | 0 |
|  | Agree (%) | 16.7 | 16.7 | 33.3 | 0 | 0 | 16.7 | 0 | 0 | 33.3 |
|  | Strongly agree (%) | 66.7 | 66.7 | 33.3 | 66.7 | 50 | 50 | 66.7 | 33.3 | 50 |
| University *n* |  | 7 | 7 | 7 | 7 | 7 | 7 | 7 | 7 | 7 |
|  | Strongly disagree (%) | 0 | 0 | 0 | 0 | 0 | 0 | 0 | 0 | 0 |
|  | Disagree (%) | 0 | 0 | 0 | 0 | 0 | 0 | 0 | 0 | 0 |
|  | Neither agree nor disagree (%) | 0 | 0 | 14.3 | 14.3 | 14.3 | 28.6 | 14.3 | 14.3 | 0 |
|  | Agree (%) | 28.6 | 28.6 | 42.9 | 28.6 | 14.3 | 42.9 | 42.9 | 28.6 | 57.1 |
|  | Strongly agree (%) | 71.4 | 71.4 | 42.9 | 57.1 | 71.4 | 28.6 | 42.9 | 57.1 | 42.9 |
| Other *n* |  | 7 | 9 | 8 | 9 | 8 | 7 | 8 | 8 | 8 |
|  | Strongly disagree (%) | 0 | 0 | 0 | 0 | 0 | 0 | 0 | 0 | 0 |
|  | Disagree (%) | 0 | 0 | 0 | 0 | 12.5 | 0 | 0 | 0 | 0 |
|  | Neither agree nor disagree (%) | 0 | 0 | 12.5 | 22.2 | 0 | 0 | 0 | 0 | 0 |
|  | Agree (%) | 0 | 11.1 | 12.5 | 11.1 | 12.5 | 14.3 | 12.5 | 25 | 25 |
|  | Strongly agree (%) | 100 | 88.9 | 75 | 66.7 | 75 | 85.7 | 87.5 | 75 | 75 |
| ***p* – value** |  | 0.06 | 0.014 | 0.534 | 0.612 | 0.508 | 0.388 | 0.510 | 0.467 | 0.367 |
| **Relatives** |  |  |  |  |  |  |  |  |  |  |
| Compulsory school *n* |  | 6 | 6 | 6 | 6 | 6 | 6 | 6 | 6 | 6 |
|  | Strongly disagree (%) | 0 | 0 | 0 | 0 | 0 | 0 | 0 | 0 | 0 |
|  | Disagree (%) | 0 | 0 | 0 | 0 | 0 | 0 | 0 | 0 | 0 |
|  | Neither agree nor disagree (%) | 0 | 0 | 0 | 0 | 0 | 0 | 0 | 0 | 16.7 |
|  | Agree (%) | 33.3 | 16.7 | 50 | 33.3 | 50 | 50 | 16.7 | 33.3 | 16.7 |
|  | Strongly agree (%) | 66.7 | 83.3 | 50 | 66.7 | 50 | 50 | 83.3 | 66.7 | 66.7 |
| Apprenticeship *n* |  | 13 | 13 | 13 | 13 | 13 | 13 | 13 | 13 | 13 |
|  | Strongly disagree (%) | 7.7 | 7.7 | 7.7 | 7.7 | 7.7 | 7.7 | 7.7 | 7.7 | 7.7 |
|  | Disagree (%) | 0 | 0 | 0 | 7.7 | 0 | 0 | 0 | 0 | 7.7 |
|  | Neither agree nor disagree (%) | 7.7 | 15.4 | 23.1 | 15.4 | 23.1 | 23.1 | 30.8 | 38.5 | 30.8 |
|  | Agree (%) | 23.1 | 30.8 | 15.4 | 23.1 | 23.1 | 30.8 | 7.7 | 0 | 15.4 |
|  | Strongly agree (%) | 61.5 | 46.2 | 53.8 | 46.2 | 46.2 | 38.5 | 53.8 | 53.8 | 38.5 |
| High school diploma *n* |  | 8 | 8 | 8 | 8 | 8 | 8 | 8 | 8 | 8 |
|  | Strongly disagree (%) | 0 | 0 | 0 | 0 | 0 | 0 | 0 | 0 | 0 |
|  | Disagree (%) | 0 | 0 | 0 | 0 | 12.5 | 0 | 0 | 12.5 | 12.5 |
|  | Neither agree nor disagree (%) | 0 | 0 | 12.5 | 12.5 | 12.5 | 12.5 | 0 | 0 | 0 |
|  | Agree (%) | 12.5 | 12.5 | 0 | 25 | 12.5 | 25 | 25 | 12.5 | 50 |
|  | Strongly agree (%) | 87.5 | 87.5 | 87.5 | 62.5 | 62.5 | 62.5 | 75 | 75 | 37.5 |
| University *n* |  | 8 | 8 | 8 | 8 | 8 | 8 | 8 | 8 | 8 |
|  | Strongly disagree (%) | 0 | 0 | 0 | 0 | 12.5 | 12.5 | 0 | 0 | 12.5 |
|  | Disagree (%) | 0 | 0 | 0 | 0 | 0 | 0 | 12.5 | 12.5 | 12.5 |
|  | Neither agree nor disagree (%) | 12.5 | 0 | 12.5 | 12.5 | 0 | 12.5 | 0 | 12.5 | 12.5 |
|  | Agree (%) | 0 | 25 | 12.5 | 37.5 | 12.5 | 0 | 12.5 | 25 | 12.5 |
|  | Strongly agree (%) | 87.5 | 75 | 75 | 50 | 75 | 75 | 75 | 50 | 50 |
| Other *n* |  | 10 | 10 | 10 | 10 | 10 | 10 | 10 | 10 | 10 |
|  | Strongly disagree (%) | 0 | 0 | 0 | 0 | 0 | 0 | 0 | 0 | 0 |
|  | Disagree (%) | 0 | 0 | 10 | 10 | 0 | 0 | 0 | 0 | 0 |
|  | Neither agree nor disagree (%) | 10 | 10 | 20 | 20 | 30 | 20 | 40 | 20 | 30 |
|  | Agree (%) | 10 | 10 | 20 | 20 | 20 | 30 | 0 | 30 | 20 |
|  | Strongly agree (%) | 80 | 80 | 50 | 50 | 50 | 50 | 60 | 50 | 50 |
| ***p* – value** |  | 0.268 | 0.318 | 0.962 | 0.672 | 0.834 | 0.618 | 0.754 | 0.701 | 0.973 |

**Supporting Information, Table 4:** Recommendations and changes of the information leaflets on fall prevention based on feedback of patients and their relatives

| Recommendations | Changes |
| --- | --- |
| Missing recommendations for relatives regarding a save environment at home. | More detailed recommendations and concrete action instructions were added to the information leaflet for relatives regarding a save environment. For example, handrails, grab bars, raised toilet seats or non-slip bathmats.  A safety checklist for more precise action recommendations for the relatives is now added to the information leaflet |
| Risk factors in the bathroom – How can you make the bathroom saver? | The bathroom is now mentioned with more detailed instruction how to make it saver. |
| Who are the experts? – Who and how can help people with fall risk. Clearer presentation of the experts. | The experts are now clearly defined as general practitioners, nurses or therapists and it is shown how they could help. |
| Bigger font | The fonts stayed the same, because it already corresponded to the recommended size of at least 12pt. But the important parts in the sentence have been made bold. |
| Shorter sentences | Sentences were checked if they are as short as possible and were shortened if necessary. A reference value of 15 words per sentence was chosen. |
| More colors | No more colors were added, but brighter and stronger colors were chosen. |
| More pictures | A picture was now added to the information leaflet for patients and relatives. |
| Difficulties regarding the “+”- symbol were mentioned. It was meant to give the reader information about the evidence of the recommendations. The information leaflets included a legend explaining the symbol. | The “+” symbol was removed to avoid confusion. |
